# Ist komplette Immunität gegen Masern bei Patienten mit rheumatischen Erkrankungen ein realistisches Ziel, und wie ist es möglicherweise zu erreichen?

**DOI:** 10.1007/s00393-020-00877-1

**Published:** 2020-09-18

**Authors:** J. Braun, U. Kiltz, U. Müller-Ladner

**Affiliations:** 1 Rheumazentrum Ruhrgebiet, Herne und Ruhr-Universität Bochum, Claudiusstr 45, 44649 Herne, Deutschland; 2 St. Elisabeth Gruppe GmbH, Herne, Deutschland; 3 Abteilung für Rheumatologie und Klinische Immunologie, Kerckhoff-Klinik, Bad Nauheim, Deutschland

**Keywords:** Impfung, Antivirale Antikörper, Masernschutzgesetz, Impfausweis, Immunsuppression, Vaccination, Anti-viral antibodies, Measles protection act, Vaccination card, Immunosuppression

## Abstract

In Deutschland kommt es immer wieder zu Masernausbrüchen. Patienten mit chronisch entzündlichen Erkrankungen werden oft immunsuppressiv behandelt. In einer aktuellen Studie zeigte sich, dass etwa 7 % dieser Patienten keinen Schutz gegen Maserninfektion haben. Das ergab sich aus der fehlenden Dokumentation im Impfausweis bzw. dem nicht vorhandenen Nachweis von protektiven Antikörpern. Die Ständige Impfkommission (STIKO) empfiehlt eine erste Impfung gegen Masern als Masern-Mumps-Röteln-Kombinationsimpfung (MMR) bei Kindern im Alter von 11–14 Monaten und eine zweite Impfung im Alter von 14–23 Monaten. Für nach 1970 geborene Erwachsene wird eine Impfung gegen Masern empfohlen, wenn diese noch nicht bzw. nur einmal gegen Masern geimpft wurden oder wenn ihr Impfstatus unklar ist. Im April 2019 hat die STIKO Anwendungshinweise zu den bei Immundefizienz empfohlenen Impfungen veröffentlicht. Seit dem 1. März 2020 besteht in Deutschland zudem eine Masernimpfpflicht.

## Abstract (en)

Measles outbreaks occur rather frequently in Germany. Patients with chronic inflammatory diseases are often treated with immunosuppressants. A recent study showed that about 7% of such patients are not protected against measles according to the lack of documentation in the vaccination card or the absence of protective antibodies. The Standing Committee on Immunization (STIKO) recommends a first vaccination against measles as a measles-mumps-rubella combined vaccination (MMR) in children aged 11–14 months and a second vaccination at 14–23 months. For adults born after 1970, vaccination against measles is recommended if they have not yet been vaccinated against measles or have only been vaccinated once against measles or if their vaccination status is unclear. In April 2019, STIKO published instructions for vaccinations recommended for immunodeficiency. Since March 1, 2020, measles vaccination have been compulsory in Germany.

In einer aktuellen Studie wurde gezeigt, dass etwa 7 % der Patienten mit chronisch entzündlichen Erkrankungen keinen Schutz gegen die stark infektiöse Masernerkrankung haben [1]. Das ergab sich aus der fehlenden Dokumentation im Impfausweis bzw. dem nicht vorhandenen Nachweis von protektiven Antikörpern. Was ist in dieser Situation zu tun, wie ist ein besserer Schutz erreichbar?

## Einleitung

Impfungen sind eine essenzielle Gesundheitsmaßnahme zur Prävention von Infektionskrankheiten [2]. Bei immungeschwächten Patienten ist die Reaktion auf die Impfung zwar geringer, aber Impfstudien haben trotzdem einen Schutzeffekt gezeigt, der zu einer Verringerung der Komplikationen, der Krankenhausaufenthalte, der Behandlungskosten und sogar der Sterblichkeit führt [3, 4].

Die für die Allgemeinbevölkerung empfohlenen inaktivierten Impfstoffe sind grundsätzlich auch bei Patienten mit Immunschwäche indiziert [2–8]. Wenn eine Beeinträchtigung der spezifischen Immunität vorliegt, ist es wahrscheinlich, dass ein eher unvollständiger Schutz durch die Impfung mit Inaktivierungsimpfstoffen induziert wird. Aber auch eine nur partielle Schutzwirkung überwiegt eindeutig das minimale Risiko, das mit der Verwendung eines inaktivierten toxoidalen oder rekombinanten Impfstoffs verbunden ist [2–8].

Lebendimpfstoffe vermehren sich im Wirt und interagieren dabei mit den Wirtszellen. Diese Impfstoffe sind zwar abgeschwächt, können aber ähnliche Symptome wie die „richtige“ Infektion durch den Krankheitserreger hervorrufen, das Ausmaß ist aber bei immunkompetenten Personen nur gering. Nichtsdestoweniger können diese Impfstoffe aber bei immunsupprimierten Patienten schwere Nebenwirkungen und Komplikationen verursachen, die selten und auch tödlich verlaufen [2–8].

## Patienten mit rheumatischen Erkrankungen

Patienten mit rheumatischen Erkrankungen werden in den letzten Jahren zunehmend mit langfristig wirkenden immunsuppressiven Medikamenten, hoch dosierten Kortikosteroiden und Biologika wie TNF-α-Antagonisten und Rituximab behandelt [9]. Patienten, die einer immunsuppressiven Behandlung bedürfen, unterscheiden sich in ihrer Immunkompetenz und dem Grad der Infektanfälligkeit. Je nach Zeitpunkt und Umfang der Therapie, der eingesetzten Medikamente kann eine Immunsuppression zu einer Beeinträchtigung von wichtigen immunologischen Funktionen führen. So wird durch den Einsatz von Biologika das Infekt- und Infektionsrisiko von Patienten mit entzündlich rheumatischen Erkrankungen erhöht [10, 11]. Zu den Empfehlungen zur Minimierung dieses Risikos gehört die Verabreichung der vollständigen Impfstoffkombination nach dem Standardimpfplan sowie anderer Impfstoffe [2–8]. Attenuierte Lebendimpfstoffe können im Prinzip erst nach einem Intervall ohne Immunsuppression und/oder Glukokortikoidtherapie verabreicht werden. Die Wirksamkeit von Impfstoffen, die auf der Grundlage von Titern schützender Antikörper bewertet wird, variiert je nach Impfstofftyp und DMARD-Therapie. So sind Methotrexat und Rituximab in der Regel mit verminderten Impfreaktionen verbunden [2–8, 12].

## Impfung mit Lebendimpfstoffen

Empfehlungen für die Impfung mit Lebendimpfstoffen von Patienten unter immunsuppressiver Behandlung beinhalten zumeist, dass Patienten, die eine tägliche bzw. hoch dosierte Glukokortikoidtherapie erhalten (≥20 mg/Tag für ≥14 Tage) nicht geimpft werden können, und das gilt für alle Lebendimpfstoffe auch 4 Wochen vor bzw. bis 3 Monate nach einer immunsuppressiven Therapie [2–8]. Grundsätzlich sollte der Impfstatus aber wegen dieser schwierigen Situation deshalb möglichst vor Therapiebeginn überprüft werden. Nach internationalen Empfehlungen sollte der Impfstatus schon bei der Erstuntersuchung von RA-Patienten bewertet und Impfstrategien sollten geplant werden, die dann idealerweise während einer stabilen Erkrankung umgesetzt werden sollten [13–15].

Obwohl bekannt ist, dass nach einer Impfung immunpathologische Reaktionen auftreten können, insbesondere bei genetisch anfälligen Wirten, deuten Untersuchungen bei RA-Patienten, die mit bDMARDs (Biologika) oder tsDMARDs (JAK-Inhibitoren) behandelt werden, darauf hin, dass Impfstoffe im Rahmen der Immunsuppression sicherer sein könnten als bisher angenommen [16–24].

Die französischen Netzwerke IREIVAC (Innovatives klinisches Forschungsnetzwerk in der Impfstoffforschung) und IMIDIATE (Allianz für immunvermittelte Entzündungskrankheiten für die transnationale und klinische Forschung) koordinieren zurzeit die Forschung auf diesen wichtigen Gebieten.

## Aktuelle Empfehlungen der ständigen Impfkommission (STIKO)

Zum Erreichen eines optimalen Schutzes vor impfpräventablen Erkrankungen sind von der STIKO vor Kurzem Anwendungshinweise für empfohlene Impfungen bei Personen mit Autoimmunkrankheiten, chronisch entzündlichen Erkrankungen und unter immunmodulatorischer Therapie gegeben worden [2–4]. Diese Hinweise wurden auf der Basis von verfügbaren klinischen Studien, internationalen Empfehlungen und den Fachinformationen der Arzneimittel und Expertenkonsens erarbeitet. Die Empfehlungen basieren auf der Feststellung, dass für Personen mit Autoimmunkrankheiten oder chronisch entzündlichen Erkrankungen ein umfassender Impfschutz essenziell ist. Im Idealfall sollte die Immunisierung vor Beginn einer immunsuppressiven Therapie bereits abgeschlossen sein (Tab. 1).

- Grundsätzlich können Totimpfstoffe bei Menschen mit einer Autoimmun- oder chronisch entzündlichen Erkrankung selbst unter immunsuppressiver Therapie angewendet werden, ohne dass ein erhöhtes Risiko für unerwünschte Wirkungen besteht.
- Obwohl der Impferfolg eingeschränkt sein kann, wird unter Therapie mit den meisten Immunsuppressiva bei einem Großteil der Patienten ein ausreichender Impfschutz erreicht – ausgenommen hiervon ist die Gabe von B‑ und/oder T‑Zell-depletierenden Antikörpern, wie z. B. Rituximab und Alemtuzumab, die zu einer fast kompletten Suppression der humoralen und/oder zellulären adaptiven Immunantwort führen.
- Dagegen sollten Lebendimpfstoffe während einer immunsuppressiven Therapie grundsätzlich nicht verabreicht werden. Dabei gibt es nur wenige Ausnahmen (z. B. niedrig dosierte Glukokortikoid- oder Methotrexat-Therapie), die jedoch eine individuelle Risiko-Nutzen-Abschätzung unter Berücksichtigung des aktuellen Immunstatus sowie eine ausführliche Patientenaufklärung erfordern. Die empfohlenen Mindestabstände für Impfungen vor bzw. nach immunsuppressiver Therapie richten sich nach dem verabreichten Medikament.

**Table 1:** 

Impfung gegen	Kategorie	Indikation	Anmerkungen (Packungsbeilage/Fachinformation beachten)
*Masern, Mumps, Röteln (MMR)*	*B*	Nach 1970 geborene Personen (einschließlich Auszubildende, PraktikantInnen, Studierende und ehrenamtlich Tätige) in folgenden Tätigkeitsbereichen: – Medizinische Einrichtungen (gemäß § 23 (3) Satz 1 IfSG) inklusive Einrichtungen sonstiger humanmedizinischer Heilberufe – Tätigkeiten mit Kontakt zu potenziell infektiösem Material – Einrichtungen der Pflege (gemäß § 71 SGB XI) – Gemeinschaftseinrichtungen (gemäß § 33 IfSG) – Einrichtungen zur gemeinschaftlichen Unterbringung von Asylbewerbern, Ausreisepflichtigen, Flüchtlingen und Spätaussiedlern – Fach‑, Berufs- und Hochschulen	Insgesamt 2‑malige Impfung mit einem MMR-Impfstoff (bei gleichzeitiger Indikation zur Varizellenimpfung ggf. MMRV-Kombinationsimpfstoff verwenden)
			Die Anzahl der notwendigen Impfstoffdosen richtet sich nach der Komponente mit den wenigsten dokumentierten Impfungen
			Bei *Frauen* ist für jede der 3 Impfstoffkomponenten (M–M–R) eine 2‑malige Impfung erforderlich
			Bei *Männern* ist für die Masern- und Mumpsimpfstoffkomponente eine 2‑malige Impfung erforderlich. Zum Schutz gegen Röteln reicht eine 1‑malige Impfung aus
			Es existieren keine Sicherheitsbedenken gegen weitere MMR-Impfung(en) bei bestehender Immunität gegen eine der Komponenten
*Varizellen*	*B*	Seronegative Personen (einschließlich Auszubildende, PraktikantInnen, Studierende und ehrenamtlich Tätige) in folgenden Tätigkeitsbereichen: – Medizinische Einrichtungen (gemäß § 23 (3) Satz 1 IfSG) inklusive Einrichtungen sonstiger humanmedizinischer Heilberufe – Tätigkeiten mit Kontakt zu potenziell infektiösem Material – Einrichtungen der Pflege (gemäß § 71 SGB XI) – Gemeinschaftseinrichtungen (gemäß § 33 IfSG) – Einrichtungen zur gemeinschaftlichen Unterbringung von Asylbewerbern, Ausreisepflichtigen, Flüchtlingen und Spätaussiedlern	Insgesamt 2‑malige Impfung (bei gleichzeitiger Indikation zur MMR-Impfung ggf. MMRV-Kombinationsimpfstoff verwenden)

Die Definition für eine niedrig dosierte Glukokortikoidtherapie ist laut Robert-Koch-Institut (RKI) [4] dabei eine Behandlung mit <10 mg Prednisolonäquivalent/Tag bei Erwachsenen bzw. 0,2 mg/kgKG bei Kindern oder eine Therapiedauer <2 Wochen oder eine nichtsystemische Glukokortikoidtherapie.

Die Anzahl behandelbarer Patientinnen und Patienten mit einer Autoimmunkrankheit oder mit einer chronisch entzündlichen Erkrankung hat in den letzten Jahren stetig zugenommen. Durch die kontinuierlich steigende Anzahl neuer immunsuppressiver Therapien, insbesondere durch den Einsatz von Biologika und Biosimilars, haben sich Prognose und Therapieerfolg von rheumatischen Erkrankungen zum Teil erheblich verbessert [25]. Deshalb werden heute zunehmend Menschen behandelt, deren Immunsystem krankheits- und/oder therapiebedingt funktionell eingeschränkt ist.

Menschen mit chronisch entzündlich rheumatischen Erkrankungen sind grundsätzlich einem erhöhten Infektionsrisiko ausgesetzt [11]. Dies ist zum Teil Folge der Erkrankung und zum Teil auf die Therapie zurückzuführen [12, 26–28]. Gegenüber nicht immunsupprimierten Patienten ist das Risiko für bestimmte virale, bakterielle, fungale oder parasitäre Infektionen unter Therapie mit Biologika um etwa das Doppelte erhöht, dabei hängt das Risiko von der Grunderkrankung und von dem jeweiligen Medikament ab [11, 26–28]. Das Risiko erhöht sich potenziell auch durch mögliche Kombinationstherapien, was auch Vortherapien z. B. mit Rituximab beinhaltet. Hier ist die biologische Halbwertszeit der Medikamente zu beachten.

Zudem können impfpräventable Infektionen wie Influenza, Varizellen/Herpes zoster, Pneumokokken, Masern oder Hepatitis B [9] bei manifester Autoimmunerkrankung oder unter immunsuppressiver Therapie schwerere Verläufe aufweisen bzw. mit einem erhöhten Risiko für Komplikationen einhergehen als bei sonst gesunden immunkompetenten Personen. Schließlich können Infektionskrankheiten wiederum einen Schub einer bestehenden Autoimmunerkrankung auslösen [29, 30].

Aufgrund der Anfälligkeit für Infektionskrankheiten können betroffene Patienten besonders von Impfungen profitieren. Allerdings kann die Wirksamkeit einer Impfung sowohl durch die Erkrankung selbst als auch durch die immunsuppressive Therapie selbst verringert werden.

Grundsätzlich wird empfohlen, vor der Durchführung einer Totimpfung bzw. auch danach einen Abstand von 2 bis 4 Wochen vor Beginn einer immunsuppressiven Therapie einzuhalten. Im Falle von Rituximab und Alemtuzumab beträgt der ideale Abstand nach deren letzter Gabe 6 Monate und davor 4 bis 6 Wochen [4].

Bei Lebendimpfungen ist die Gesundheit von Geimpften durch den attenuierten Impferreger grundsätzlich als gefährdet anzusehen [31–34]. Deshalb ist bei Lebendimpfungen eine genaue Nutzen-Risiko-Abschätzung essenziell.

Die Möglichkeit einer serologischen Kontrolle des Impferfolgs wie z. B. bei Tetanus und Diphterie sollte bei entsprechender Fragestellung in Betracht gezogen werden.

Die Impfquoten von Menschen mit Autoimmunkrankheiten und anderen chronisch entzündlichen Erkrankungen bzw. unter immunmodulatorischer Therapie sind oft geringer als bei Gesunden [35]. Patienten unter Immunsuppression werden aus Sorge vor mangelnder Sicherheit und unzureichender Wirksamkeit der Impfungen häufig nicht ausreichend geimpft [36]. Das hat einen fehlenden Schutz vor vermeidbaren Risiken zur Folge.

Für keinen der derzeit in Deutschland zugelassenen Tot- oder Lebendimpfstoffe existieren Studien, die einen ursächlichen Zusammenhang zwischen der Impfung und einer neu aufgetretenen Autoimmunkrankheit bzw. chronisch entzündlichen Erkrankung oder einem Schub einer solchen bereits bestehenden Erkrankung belegen [3, 37].

## Die Masern-Mumps-Röteln-Impfung (MMR)

Die MMR-Impfung ist für Menschen mit schwerer Immundefizienz laut Fachinformation generell kontraindiziert [38]. Die altersentsprechende Grundimmunisierung nach den Empfehlungen der STIKO sollte daher möglichst vor Einleitung einer immunsuppressiven Therapie abgeschlossen sein. Wurde vor Beginn der Therapie in der Kindheit nur einmalig gegen MMR geimpft, sollte die Komplettierung der Impfserie altersentsprechend abgeschlossen werden. Wenn eine Impfung nicht möglich ist und anhand des Impfpasses oder der ärztlichen Dokumentation nicht von einem Schutz ausgegangen werden kann, sollte eine serologische Kontrolle erfolgen, die Aufschluss darüber geben kann, ob bereits ein ausreichender Schutz besteht.

Eine niedrig dosierte Glukokortikoidtherapie (s. Definition) stellt für die verfügbaren MMR-Impfstoffe keine Kontraindikation dar [4]. Das gilt laut Fachinformation im Falle einer geringgradigen Immunsuppression auch für Priorix® (MMR-Impfung) bzw. PriorixTetra® (MMR-V-Impfung) – im Gegensatz zu M‑M-RVaxPro® bzw. ProQuad®. Die erstgenannten Impfstoffe können im Einzelfall bei geringgradiger Immunsuppression nach individueller Nutzen-Risiko-Abschätzung und sorgfältiger Aufklärung verwendet werden ([4], Off-label-Gebrauch). Einige kleine Studien bei juveniler idiopathischer Arthritis (JIA) weisen auf vorhandene Sicherheit und Effektivität der MMR-Impfung hin (s. oben; [17, 18]).

## Das neue Masernschutzgesetz

Am 01.03.2020 ist das neue Masernschutzgesetz in Kraft getreten, welches eine Impflicht gegen Masern beinhaltet [39]. Auf dieser Basis hat die STIKO vor Kurzem ihre Empfehlungen zur beruflichen Indikation der Masern‑, Mumps- und Rötelnimpfungen aus gegebenem Anlass (Masernschutzgesetz) aktualisiert [40, 41]. Die Empfehlungen zur Impfung gegen diese 3 Erreger aufgrund beruflicher Tätigkeit wurden nunmehr in einer Empfehlung zur beruflich indizierten Impfung gegen Masern, Mumps und Röteln (MMR) zusammengefasst und in Bezug auf die Indikationsgruppen unter Berücksichtigung der vorliegenden Evidenz angeglichen. Die MMR-Impfung ist demnach für nach 1970 geborene Personen (einschließlich Auszubildende, PraktikantInnen, Studierende und ehrenamtlich Tätige) in folgenden beruflichen Tätigkeitsbereichen indiziert (Auszug):

- medizinische Einrichtungen (gemäß § 23 Absatz 3 Satz 1 Infektionsschutzgesetz [IfSG])
- inklusive Einrichtungen sonstiger humanmedizinischer Heilberufe,
- Tätigkeiten mit Kontakt zu potenziell infektiösem Material,
- Einrichtungen der Pflege (gemäß § 71 SGB XI).

Damit müssen alle in der Rheumatologie tätigen Ärzte und Pflegekräfte, die nach 1970 geboren wurden, gegen Masern geimpft sein, und der Träger der Einrichtung bzw. der leitende Arzt oder Betriebsarzt wird sich davon überzeugen müssen, dass das auch geschehen ist bzw. dass ein Schutz besteht.

Grundsätzlich gilt nach dem Masernschutzgesetz:

- Zur Durchführung von Schutzimpfungen ist jeder Arzt berechtigt. Fachärzte dürfen Schutzimpfungen unabhängig von den Grenzen der Ausübung ihrer fachärztlichen Tätigkeit durchführen.
- Jede Schutzimpfung ist unverzüglich in einen Impfausweis, oder, falls der Impfausweis nicht vorgelegt wird, in einer Impfbescheinigung zu dokumentieren (Impfdokumentation).
- Die Impfdokumentation muss zu jeder Schutzimpfung folgende Angaben enthalten:
  1. Datum der Schutzimpfung,
  2. Bezeichnung und Chargenbezeichnung des Impfstoffes,
  3. Name der Krankheit, gegen die geimpft wurde.
- Ein ausreichender Impfschutz gegen Masern besteht, wenn ab der Vollendung des ersten Lebensjahres mindestens 1 Schutzimpfung und ab der Vollendung des zweiten Lebensjahres mindestens 2 Schutzimpfungen gegen Masern bei der betroffenen Person durchgeführt wurden.
- Personen, die aufgrund einer medizinischen Kontraindikation nicht an Schutzimpfungen oder an anderen Maßnahmen der spezifischen Prophylaxe teilnehmen können, können durch Rechtsverordnung nicht zu einer Teilnahme an Schutzimpfungen oder an anderen Maßnahmen der spezifischen Prophylaxe verpflichtet werden.

Personen, die in Gemeinschaftseinrichtungen nach § 33 wie in Krankenhäusern tätig werden sollen, haben der Leitung der jeweiligen Einrichtung vor Beginn ihrer Betreuung oder ihrer Tätigkeit folgenden Nachweis vorzulegen:

1. eine Impfdokumentation nach § 22 oder ein ärztliches Zeugnis, auch in Form einer Dokumentation nach § 26 SGB 5, darüber, dass bei ihnen nach den entsprechenden Maßgaben ausreichender Impfschutz gegen Masern besteht,
2. ein ärztliches Zeugnis darüber, dass bei ihnen eine Immunität gegen Masern vorliegt oder sie aufgrund einer medizinischen Kontraindikation nicht geimpft werden können, oder
3. eine Bestätigung einer staatlichen Stelle oder der Leitung einer anderen Einrichtung darüber, dass ein Nachweis bereits vorgelegen hat.

Wenn der Impfnachweis von einer Person, die dazu verpflichtet ist, nicht vorgelegt wird oder wenn sich ergibt, dass ein Impfschutz gegen Masern erst zu einem späteren Zeitpunkt möglich ist oder vervollständigt werden kann, hat

1. die Leitung der jeweiligen Einrichtung oder
2. die andere Stelle unverzüglich das Gesundheitsamt, in dessen Bezirk sich die Einrichtung befindet, zu benachrichtigen und dem Gesundheitsamt personenbezogene Angaben zu übermitteln.

Nach den Vorgaben des RKI sollte die Kontrolle der Masernimmunität grundsätzlich durch Kontrolle des Impfausweises erfolgen [41]. Danach ist nur, wenn mindestens 2 MMR- oder Masernimpfungen im Impfpass dokumentiert sind, davon auszugehen, dass die Passinhaber zuverlässig gegen Masern geschützt sind. Der Abstand zwischen 2 Masernimpfungen sollte mindestens 4 Wochen betragen.

Eine Antikörperkontrolle ist in diesem Fall laut RKI normalerweise nicht erforderlich und wird nicht empfohlen [41].

Allerdings sagt das RKI auch klar, dass bei positivem Nachweis von Anti-Masern-IgG-Antikörpern grundsätzlich von Immunität, d. h. von Schutz gegen Masern, ausgegangen werden kann [41].

## Daher schlagen wir für Patienten mit rheumatischen Erkrankungen und laufender immunsuppressiver Medikation die folgende Vorgehensweise vor

1. Die Patienten, die vor 1970 geboren sind, werden über das Gesetz informiert und gebeten, den Impfausweis vorzulegen.
2. Wenn 2 Masernimpfungen dokumentiert sind, ist von Immunität auszugehen, und keine weiteren Maßnahmen sind erforderlich.
3. Wenn sich der Patient eindeutig erinnert, eine Maserninfektion gehabt zu haben, kann ebenfalls Immunität angenommen werden. Zur Bestätigung ist eine IgG-Antikörperbestimmung gegen Masern sinnvoll, v. a. in unklaren Fällen.
4. Wenn weder 2 Masernimpfungen dokumentiert sind noch eine Maserninfektion erinnert wird, sollte in jedem Fall eine IgG-Antikörperbestimmung gegen Masern veranlasst werden.
5. Im Falle eines IgG-Antikörpernachweises gegen Masern ist ebenfalls von Immunität auszugehen. Dies ist in über 90 % der Fälle zu erwarten.
6. Wenn weder 2 Masernimpfungen dokumentiert sind noch eine Maserninfektion erinnert wird noch ein Nachweis von IgG-Antikörpern geführt werden konnte, sollte geprüft werden, ob die immunsuppressive Therapie pausiert werden kann, um eine Masernimpfung durchzuführen.
7. Wenn das nicht möglich ist, muss es entsprechend dokumentiert werden.
8. Der Impfschutz aller Patienten sollte initial bei der Diagnosestellung überprüft und ggf. durch Impfung korrigiert werden.

Der empfohlene Mindestabstand zwischen 2 Masernimpfungen beträgt ≥4 Wochen. Bei der Impfung im Alter von 6 bis 8 Monaten sind insgesamt 3 Impfungen zum Aufbau einer Langzeitimmunität notwendig.

Zusammengefasst haben Menschen mit Autoimmunkrankheiten, chronisch entzündlichen Erkrankungen und/oder unter immunmodulatorischer Therapie ein erhöhtes Infektionsrisiko. Bei nicht geimpften Personen mit Autoimmunkrankheiten oder chronisch entzündlichen Erkrankungen können Infektionen, die durch eine Impfung verhindert werden könnten, Morbidität und Mortalität erhöhen. Impfungen mit in Deutschland zugelassenen Tot- oder Lebendimpfstoffen lösen keine Schübe rheumatischer Erkrankungen aus, können aber im Gegenteil das Risiko für infektionsgetriggerte Schübe verringern.

Insgesamt ist festzuhalten, dass der Impfschutz gegen Masern bei Patienten mit rheumatischen Erkrankungen noch unzureichend ist. Obwohl es bisher keine Berichte über eine größere Zahl von Masernfällen bei diesen Patienten gibt, ist ein sicherer Impfschutz auch unter dem Aspekt einer ausreichenden Herdenimmunität zu gewährleisten. Die neue Gesetzeslage ermöglicht es und verpflichtet die Rheumatologen, dafür Sorge zu tragen, dass möglichst alle Patienten mit rheumatischen Erkrankungen gegen Masern geschützt sind. Das wird nicht für alle Patienten möglich sein, denn v. a. die Patienten mit persistierend hoher Krankheitsaktivität und Immunsuppression wird man nicht parallel impfen können. Deshalb stellt die möglichst frühe Impfung am besten noch vor Beginn der Erkrankung oder früh im Krankheitsverlauf sicher die beste Strategie dar.

## References

[1] Kiltz U, Celik A, Tsiami S, Baraliakos X, Andreica I, Kiefer D, Bühring B, Braun J (2020). Wie gut sind Patienten mit entzündlich rheumatischen Erkrankungen gegen Masern geschützt?. Z Rheumatol.

[2] RKI (2018). Empfehlungen der Ständigen Impfkommission (STIKO) am Robert Koch-Institut. Epidemiol Bull.

[3] Niehues T, Bogdan C, Hecht J, Mertens T, Wiese-Posselt M, Zepp F (2017). Impfen bei Immundefizienz: Anwendungshinweise zu den von der Ständigen Impfkommission empfohlenen Impfungen (I) Grundlagenpapier. Bundesgesundheitsblatt Gesundheitsforschung Gesundheitsschutz.

[4] Wagner N, Assmus F, Arendt G, Baum E, Baumann U, Bogdan C, Burchard G, Föll D, Garbe E, Hecht J, Müller-Ladner U, Niehues T, Überla K, Vygen-Bonnet S, Weinke T, Wiese-Posselt M, Wojcinski M, Zepp F (2019). Impfen bei Immundefizienz: Anwendungshinweise zu den von der Ständigen Impfkommission empfohlenen Impfungen. (IV) Impfen bei Autoimmunkrankheiten, bei anderen chronisch-entzündlichen Erkrankungen und unter immunmodulatorischer Therapie. Bundesgesundheitsblatt Gesundheitsforschung Gesundheitsschutz.

[5] Shearer WT, Fleisher TA, Buckley RH (2014). Recommendations for live viral and bacterial vaccines in immunodeficient patients and their close contacts. J Allergy Clin Immunol.

[6] Eibl MM, Wolf HM (2015). Vaccination in patients with primary immune deficiency, secondary immune deficiency and autoimmunity with immune regulatory abnormalities. Immunotherapy.

[7] Morel J, Czitrom SG, Mallick A, Sellam J, Sibilia J (2016). Vaccinations in adults with chronic inflammatory joint disease: immunization schedule and recommendations for patients taking synthetic or biological disease-modifying antirheumatic drugs. Joint Bone Spine.

[8] Lopez A, Mariette X, Bachelez H (2017). Vaccination recommendations for the adult immunosuppressed patient: a systematic review and comprehensive field synopsis. J Autoimmun.

[9] Hua C, Barnetche T, Combe B, Morel J (2014). Effect of methotrexate, anti-tumor necrosis factor α, and rituximab on the immune response to influenza and pneumococcal vaccines in patients with rheumatoid arthritis: a systematic review and meta-analysis. Arthritis Care Res (Hoboken).

[10] Aga AB, Lie E, Uhlig T (2015). Time trends in disease activity, response and remission rates in rheumatoid arthritis during the past decade: results from the NOR-DMARD study 2000–2010. Ann Rheum Dis.

[11] Doran MF, Crowson CS, Pond GR, O’Fallon WM, Gabriel SE (2002). Frequency of infection in patients with rheumatoid arthritis compared with controls: a population-based study. Arthritis Rheum.

[12] Listing J, Kekow J, Manger B (2015). Mortality in rheumatoid arthritis: the impact of disease activity, treatment with glucocorticoids, TNFα inhibitors and rituximab. Ann Rheum Dis.

[13] Furer V, Rondaan C, Heijstek M, van Assen S, Bijl M, Agmon-Levin N, Breedveld FC, D’Amelio R, Dougados M, Kapetanovic MC, van Laar JM, Ladefoged de Thurah A, Landewé R, Molto A, Müller-Ladner U, Schreiber K, Smolar L, Walker J, Warnatz K, Wulffraat NM, Elkayam O (2019). Incidence and prevalence of vaccine preventable infections in adult patients with autoimmune inflammatory rheumatic diseases (AIIRD): a systemic literature review informing the 2019 update of the EULAR recommendations for vaccination in adult patients with AIIRD. RMD Open.

[14] Rondaan C, Furer V, Heijstek MW, Agmon-Levin N, Bijl M, Breedveld FC, D’Amelio R, Dougados M, Kapetanovic MC, van Laar JM, Ladefoged de Thurah A, Landewé R, Molto A, Müller-Ladner U, Schreiber K, Smolar L, Walker J, Warnatz K, Wulffraat NM, van Assen S, Elkayam O (2019). Efficacy, immunogenicity and safety of vaccination in adult patients with autoimmune inflammatory rheumatic diseases: a systematic literature review for the 2019 update of EULAR recommendations. RMD Open.

[15] Furer V, Rondaan C, Heijstek MW, Agmon-Levin N, van Assen S, Bijl M, Breedveld FC, D’Amelio R, Dougados M, Kapetanovic MC, van Laar JM, de Thurah A, Landewé RB, Molto A, Müller-Ladner U, Schreiber K, Smolar L, Walker J, Warnatz K, Wulffraat NM, Elkayam O (2020). 2019 update of EULAR recommendations for vaccination in adult patients with autoimmune inflammatory rheumatic diseases. Ann Rheum Dis.

[16] Ljungman P, Fridell E, Lönnqvist B, Bolme P, Böttiger M, Gahrton G, Linde A, Ringdén O, Wahren B (1989). Efficacy and safety of vaccination of marrow transplant recipients with a live attenuated measles, mumps, and rubella vaccine. J Infect Dis.

[17] Borte S, Liebert UG, Borte M, Sack U (2009). Efficacy of measles, mumps and rubella revaccination in children with juvenile idiopathic arthritis treated with methotrexate and etanercept. Rheumatology (Oxford).

[18] Heijstek MW, Kamphuis S, Armbrust W, Swart J, Gorter S, de Vries LD, Smits GP, van Gageldonk PG, Berbers GA, Wulffraat NM (2013). Effects of the live attenuated measles-mumps-rubella booster vaccination on disease activity in patients with juvenile idiopathic arthritis: a randomized trial. JAMA.

[19] Kawano Y, Suzuki M, Kawada J, Kimura H, Kamei H, Ohnishi Y, Ono Y, Uchida H, Ogura Y, Ito Y (2015). Effectiveness and safety of immunization with live-attenuated and inactivated vaccines for pediatric liver transplantation recipients. Vaccine.

[20] Del Porto F, Laganà B, Biselli R, Donatelli I, Campitelli L, Nisini R, Cardelli P, Rossi F, D’Amelio R (2006). Influenza vaccine administration in patients with systemic lupus erythematosus and rheumatoid arthritis. Safety and immunogenicity. Vaccine.

[21] Holvast A, Huckriede A, Wilschut J (2006). Safety and efficacy of influenza vaccination in systemic lupus erythematosus patients with quiescent disease. Ann Rheum Dis.

[22] Huber F, Ehrensperger B, Hatz C, Chappuis F, Bühler S, Eperon G (2018). Safety of live vaccines on immunosuppressive or immunomodulatory therapy-a retrospective study in three Swiss Travel Clinics. J Travel Med.

[23] Cagol L, Seitel T, Ehrenberg S, Frivolt K, Krahl A, Lainka E, Gerner P, Lenhartz H, Vermehren J, Radke M, Trenkel S, Mayer B, Koletzko S, Debatin KM, Mertens T, Posovszky C (2020). Vaccination rate and immunity of children and adolescents with inflammatory bowel disease or autoimmune hepatitis in Germany. Vaccine.

[24] Uziel Y, Moshe V, Onozo B, Kulcsár A, Tróbert-Sipos D, Akikusa JD, Salviato Pileggi G, Maritsi D, Kasapcopur O, Rodrigues M, Smerla R, Rigante D, Makay B, Atsali E, Wulffraat N, Toplak N (2020). Live attenuated MMR/V booster vaccines in children with rheumatic diseases on immunosuppressive therapy are safe: multicenter, retrospective data collection. Vaccine.

[25] Lacaille D, Avina-Zubieta JA, Sayre EC, Abrahamowicz M (2017). Improvement in 5-year mortality in incident rheumatoid arthritis compared with the general population—Closing the mortality gap. Ann Rheum Dis.

[26] Lahiri M, Dixon W (2015). Risk of infection with biologic antirheumatic therapies in patients with rheumatoid arthritis. Best Pract Res Clin Rheumatol.

[27] Winthrop KL, Mariette X, Silva JT (2018). ESCMID Study Group for Infections in Compromised Hosts (ESGICH) Consensus Document on the safety of targeted and biological therapies: an infectious diseases perspective (soluble immune effector molecules [II]: agents targeting interleukins, immunoglobulins and complement factors). Clin Microbiol Infect.

[28] McKinnon JE, Maksimowicz-McKinnon K (2016). Autoimmune disease and vaccination: impact on infectious disease prevention and a look at future applications. Transl Res.

[29] Oikonen M, Laaksonen M, Aalto V (2011). Temporal relationship between environmental influenza A and Epstein-Barr viral infections and high multiple sclerosis relapse occurrence. Mult Scler.

[30] De Keyser J, Zwanikken C, Boon M (1998). Effects of influenza vaccination and influenza illness on exacerbations in multiple sclerosis. J Neurol Sci.

[31] Bakare N, Menschik D, Tiernan R, Hua W, Martin D (2010). Severe combined immunodeficiency (SCID) and rotavirus vaccination: reports to the Vaccine Adverse Events Reporting System (VAERS). Vaccine.

[32] Morillo-Gutierrez B, Worth A, Valappil M, Gaspar HB, Gennery AR (2015). Chronic infection with Rotavirus vaccine strains in UK children with severe combined Immunodeficiency. Pediatr Infect Dis J.

[33] Neven B, Perot P, Bruneau J (2017). Cutaneous and visceral chronic granulomatous disease triggered by a rubella virus vaccine strain in children with primary Immunodeficiencies. Clin Infect Dis.

[34] Perelygina L, Plotkin S, Russo P (2016). Rubella persistence in epidermal keratinocytes and granuloma M2 macrophages in patients with primary immunodeficiencies. J Allergy Clin Immunol.

[35] Loubet P, Kerneis S, Groh M (2015). Attitude, knowledge and factors associated with influenza and pneumococcal vaccine uptake in a large cohort of patients with secondary immunodeficiency. Vaccine.

[36] Hmamouchi I, Winthrop K, Launay O, Dougados M (2015). Low rate of influenza and pneumococcal vaccine coverage in rheumatoid arthritis: data from the international COMORA cohort. Vaccine.

[37] Confavreux C, Suissa S, Saddier P, Bourdes V, Vukusic S (2001). Vaccinations and the risk of relapse in multiple sclerosis. Vaccines in multiple sclerosis study group. N Engl J Med.

[38] RKI (2020) Epidemiologisches Bulletin. https://www.rki.de/DE/Content/Infekt/EpidBull/Archiv/2020/Ausgaben/02_20.pdf?__blob=publicationFile. Zugegriffen: 15.6.2020

[39] Gesetz für den Schutz vor Masern und zur Stärkung der Impfprävention (Masernschutzgesetz) vom 10. Februar 2020. Bundesgesetzblatt Jahrgang 2020 Teil I Nr. 6, ausgegeben zu Bonn am 13. Februar 2020

[40] RKI (2015) Antworten auf häufig gestellte Fragen (FAQ) zum Thema Masern. Stand: 23.02.2015. https://www.rki.de/SharedDocs/FAQ/MMR/Masern/Liste_Masern.html. Zugegriffen: 15.6.2020

[41] RKI (2020). Mitteilung der Ständigen Impfkommission beim Robert Koch-Institut: Empfehlung und wissenschaftliche Begründung für die Angleichung der beruflich indizierten Masern-Mumps-Röteln-(MMR-) und Varizellen-Impfung. Epidemiol Bull.

